# Effectiveness of internet-based CBT-I for the treatment of chronic subthreshold to moderate insomnia

**DOI:** 10.3389/fneur.2023.1180339

**Published:** 2023-06-02

**Authors:** Wongpitak Theppornpitak, Solaphat Hemrungrojn, Krittapas Thienwiwatnukul, Dittapol Muntham, Naricha Chirakalwasan, Puntarik Srisawart

**Affiliations:** ^1^Excellence Center for Sleep Disorders, King Chulalongkorn Memorial Hospital, Bangkok, Thailand; ^2^Department of Psychiatry, Faculty of Medicine, Chulalongkorn University, Bangkok, Thailand; ^3^Cognitive Fitness and Biopsychological Technology Research Unit, Chulalongkorn University, Bangkok, Thailand; ^4^Section for Mathematics, Faculty of Science and Technology, Rajamangala University of Technology Suvarnabhumi, Phranakhon Si Ayutthaya, Thailand; ^5^Division of Pulmonary and Critical Care Medicine, Department of Medicine, Faculty of Medicine, Chulalongkorn University, Bangkok, Thailand

**Keywords:** chronic insomnia, cognitive-behavioral therapy for insomnia, internet-based CBT-I, chronic subthreshold to moderate insomnia, sleep diary

## Abstract

**Study objectives:**

To study the effectiveness of the first internet-based cognitive behavioral therapy for insomnia (CBT-i) in Thailand, using the Nitra application, for chronic subthreshold to moderate insomnia treatment.

**Methods:**

An interventional study without a control group was conducted between January and June 2022. Participants were adults aged 18 years old and older with subthreshold to moderate severity of chronic insomnia (insomnia severity index (ISI) of 8–21) and had mean sleep efficiency <85% from baseline sleep diaries. Baseline sleep characteristics were obtained from questionnaires and sleep diaries from the Nitra application for 2 weeks. Eligible participants continued using the Nitra application for 4 weeks during the intervention period. Interventions including sleep restriction, stimulus control, cognitive restructuring, relaxation techniques, and sleep hygiene education were implemented *via* the pre-programmed Nitra application. Post-intervention sleep characteristics were also obtained from questionnaires and sleep diaries from the Nitra application for another 1 week.

**Results:**

A total of 40 participants completed the study. All participants had a baseline sleep efficiency of less than 85% with the majority of the participants having a sleep-onset insomnia problem (98%). For the primary outcome, sleep efficiency was significantly improved after using the Nitra application (*p* < 0.001). Self-reported total sleep time, sleep onset latency, wake after sleep onset, early morning awakening, ISI, Pittsburgh Sleep Quality Index (PSQI), and average subjective sleep quality were also significantly improved (*p* < 0.001 for all parameters except *p* = 0.017 for total sleep time and *p* = 0.018 for wake after sleep onset). Participants who had a low baseline ISI and went to bed and woke up within 30 minutes of a designated bedtime and wake-up time recommended by the Nitra application for ≥70% of all nights demonstrated an increased chance of achieving normal sleep efficiency after using the Nitra application.

**Conclusion:**

This first internet-based CBT-i in Thailand, using the Nitra application, effectively improved sleep efficiency and other sleep parameters in chronic subthreshold to moderate insomnia.

## 1. Introduction

Insomnia is a common problem among the Thai population. The study by Sukying C in Thailand found that nearly half of the elderly population, who were aged more than 60 years old, had insomnia problems (1). Insomnia can impair the daytime function of the patients and cause a high economic burden related to direct costs (consultation and treatment of insomnia) and indirect costs (reduced productivity) (2). Benzodiazepines, as hypnotic medications, are the most commonly prescribed drug to patients with insomnia (3). However, there is evidence concerning the long-term use of these medications of the risk of dependence and tolerance over time and adverse effects such as daytime drowsiness, motor vehicle accidents, falling, and increased risk of developing dementia (4). Currently, the recommended standard treatment for chronic insomnia is cognitive behavioral therapy for insomnia (CBT-i) (5). This treatment is a multimodal intervention that includes sleep restriction, stimulus control, cognitive restructuring, relaxation techniques, and sleep hygiene education (6). Many studies have shown that CBT-i significantly improved patient’s insomnia, and this effect was sustained in the long term, without adverse effects (7–9). However, due to the few available CBT-i providers, the frequency and long duration of each visit, and the requirement of hospital visits, access to CBT-i for many patients is limited.

However, with the generalized accessibility of internet technology, CBT-i can be delivered to many patients *via* the internet. Many studies showed that internet-based CBT-i could improve patient’s insomnia and the effect was sustained in the long term like traditional CBT-i (10–13). However, some studies showed that internet-based CBT-i may not be as effective as traditional face-to-face CBT-I but it is still better than a placebo (14). Given the advantage of internet-based CBT-i in terms of more availability, more accessibility, and relatively lower costs, implementation of this treatment modality for insomnia seems to be appropriate. However, there is no currently available published literature examining the use of both face-to-face and internet-based CBT-i in Thailand. This study aims to evaluate the effectiveness of the first internet-based CBT-i in Thailand using the innovative Nitra application for the treatment of insomnia.

## 2. Materials and methods

### 2.1. Study design

This study was conducted as an interventional study without a control group to determine the effectiveness of internet-based CBT-i using the Nitra application for insomnia treatment in the Thai population.

### 2.2. Participants

Recruitment of participants was conducted between January 2022 and June 2022. A poster with information about the study was advertised to the community *via* the official online website and the Facebook page of the Excellence Center for Sleep Disorders, King Chulalongkorn Memorial Hospital, Thai Red Cross Society, Bangkok, Thailand, and by a local poster published in the hospital. A total of 249 individuals with insomnia showed interest in participating in the study and applied *via* a Google Form linked to the QR code shown in the advertised poster. Telephone interviews, multiple questionnaires, and sleep diaries collected *via* the Nitra application were used to screen the potential research subjects using the inclusion and exclusion criteria. The inclusion criteria for participants included being at least 18 years old, having a clinical diagnosis of chronic insomnia disorders based on International Classification of Sleep Disorders, Version 3 (ICSD-3) criteria, with subthreshold to moderate severity of insomnia problem (insomnia severity index (ISI) of 8–21) and were able to access the Nitra application *via* a web browser on a smartphone, tablet, or computer. Since this application was the first internet-based CBT-i study in Thailand, severe insomnia patients, who would benefit from standard face-to-face CBT-i treatment, were not included in our study. The exclusion criteria included a history or high risk of other sleep disorders (obstructive sleep apnea, restless legs syndrome, or narcolepsy) or psychiatric disorders (bipolar disorder, major depressive disorder, anxiety disorder, or post-traumatic stress disorder), evaluated by standard questionnaires, namely STOP-Bang (15), IRLSSG (International Restless Legs Syndrome Study Group) questionnaire (16), ESS (Epworth Sleepiness Scale) (17), GAD-7 (Generalized anxiety disorder-7) (18), PHQ-9 (Patient Health Questionnaire-9) (19), and PTSD-5 (Posttraumatic stress disorder-5) (20). The participants would be considered high risk for obstructive sleep apnea, restless legs syndrome, narcolepsy, major depressive disorder, anxiety disorder, or post-traumatic stress disorder if they had STOP-Bang ≥5, IRLSSG = 4, ESS > 10 with at least 1 symptom of narcolepsy (sleep paralysis, hypnagogic or hypnopompic hallucination, or cataplexy), PHQ-9 ≥ 10, GAD-7 ≥ 10, or PTSD-5 ≥ 4, respectively. The participants who had recorded a mean sleep efficiency ≥85% in sleep diaries for 2 weeks were excluded. Information on each participant was collected by multiple questionnaires and sleep diaries recorded through the Nitra application. This information included baseline characteristics (age, sex, marital status, educational level, household salary, hypnotic medication use, caffeine use, alcohol use, and smoking) and sleep characteristics (circadian type by morningness-eveningness questionnaire (MEQ), ISI, Dysfunctional Beliefs and Attitudes about Sleep (DBAS), sleep quality by Pittsburgh Sleep Quality Index (PSQI), and sleep parameters by sleep diary record). All the participants who met the eligible criteria continued using the Nitra application for another 5 weeks (4 weeks of intervention period using the Nitra application and 1 week of post-intervention evaluation). Finally, a total of 40 participants were enrolled in the study.

The Nitra application was developed by the team at the Excellence Center for Sleep Disorders, which was led by the authors of this study. The Nitra application is the first fully automated internet-based CBT-i developed in Thailand. During the 4-week intervention period, each participant had to input the data for the sleep diaries in the application every day and was encouraged to take part in the sleep education provided in the application. The Nitra application provided multimodal techniques to each participant including sleep restriction, stimulus control, cognitive restructuring, relaxation technique, and sleep hygiene education. For sleep restriction, the Nitra application was pre-programmed to provide a designated bedtime and wake-up time for each week based on the data of average sleep efficiency, average total sleep time, bedtimes, and wake-up times recorded during the previous week by each participant. A preferable wake-up time was also selected by the participants. Information about stimulus control, cognitive restructuring, relaxation techniques, and sleep hygiene education was provided to each participant through short articles, some with video clips embedded, in the Nitra application. Access to the articles by each participant was also recorded by the application when the participant manually clicked the button at the end of the article to confirm the finish of the reading. Good compliance with the sleep restriction technique was defined as the participant’s bedtime and the wake-up time being within 30 minutes of the designated bedtime and wake-up time recommended by Nitra application for ≥70% of all nights. The cut point of 70% was used because this value implied that compliance was equal to 5 out of 7 days of a week. Good adherence to sleep education was defined as reading ≥50% of all articles provided by the application.

The sleep characteristics of each participant were evaluated again after completing the 4-week intervention period using the Nitra application. This information included ISI, sleep quality by PSQI, and sleep parameters recorded in the sleep diary for 1 week in the Nitra application.

### 2.3. Primary and secondary outcomes

The primary outcome was the change in sleep efficiency after using the Nitra application. The secondary outcomes included the change of other sleep parameters (total sleep time, sleep onset latency, wake after sleep onset, early morning awakening, time in bed, ISI, ESS, PSQI, and subjective sleep quality) after using the Nitra application, the percentage of participants achieving the normal value for each of the sleep parameters [sleep efficiency ≥85% (21), total sleep time ≥ 7 hours (22), sleep onset latency ≤30 minutes, wake after sleep onset ≤30 minutes, early morning awakening ≤30 minutes (21), ISI < 8 (23), and PSQI <6 (24)] after using the Nitra application, and factors associated with the improvement in sleep efficiency or achievement of normal sleep efficiency after using the Nitra application.

### 2.4. Statistical analysis

A sample size calculation was conducted using the data from the previous study by Zachariae R (12). The predicted sample size of 40 participants would provide a two-sided alpha level of 0.05 and a 90% power to detect a mean difference of 0.58 effect size of sleep efficiency change comparing between before and after using the Nitra application. This estimation assumed an approximately 20% loss to follow-up. The statistical analysis was as follows. For continuous variables, the data were reported as mean and standard deviation (normal distribution data), and median and interquartile range (non-normal distribution data). A paired *t*-test and Wilcoxon signed-rank test were used to compare dependent variables for normal distribution and non-normal distribution data, respectively. To compare independent variables, an unpaired *t*-test and Wilcoxon rank-sum test were used for parametric and non-parametric data, respectively. For categorical variables, the data were reported as counts and percentages. Comparison between dependent variables was done using McNemar’s test and comparison between independent variables was done using a chi-square test or Fisher’s exact test. A linear regression model was used to evaluate factors associated with sleep efficiency change after using the Nitra application and logistic regression models were used to evaluate factors associated with achieving normal sleep efficiency after using the Nitra application. Missing data was imputed using the data from the days before and after. Statistical analysis was performed using SPSS version 22. This study was approved by the ethics committee and registered at www.clinicaltrials.in.th (#TCTR20210901001). This research was financially supported by the Ratchadaphiseksomphot Endowment Fund of Chulalongkorn University.

## 3. Results

A total of 249 participants with insomnia showed interest in the study and applied *via* a Google Forms submission. Nine participants were unable to be contacted by telephone call and were excluded. Furthermore, 70 participants were excluded by telephone interviewing due to the following reasons: age < 18 years old, frequency of insomnia symptoms was less than 3 times/week, insomnia symptoms lasted less than 3 months, ISI was <8 or > 21, and history of comorbid sleep or psychiatric disorders. The remaining 170 participants were asked to register for the Nitra application and answer the screening questionnaires and record sleep diaries for 2 weeks. During this step, 125 participants were excluded: 25 participants did not register for the Nitra application, four participants registered for the Nitra application but did not answer the screening questionnaires, 88 participants met the exclusion criteria from the screening questionnaire, and eight participants had a mean sleep efficiency recorded during the first 2 weeks of ≥85%. A total of 45 participants were enrolled in this study with five participants requested to stop using the application during the study period. The remaining 40 participants completed the study.

From the total of 40 participants, the average age was 45 years old and most participants were female (77.5%) with a high educational level (90% finished a bachelor’s degree or higher). From the MEQ test, the intermediate type was the most common (75%), followed by the evening type (9%), and only one participant was a morning type. More than half of the participants had a history of caffeine use (62.5%) and nearly all the participants did not use alcohol or smoke (92.5 and 97.5%, respectively). Only five participants currently used sedative medications for their insomnia problem. The average DBAS score was 99.6 from the maximum score of 160, which represented a trend of false belief and attitude about sleep (Table 1).

**Table 1:** Baseline characteristics.

Parameter	Participants (*n* = 40)	
	Mean ± S.D.	*n* (%)
Age	45.75 ± 12.74	–
Sex		
Male	–	9 (22.5)
Female	–	31 (77.5)
Marital status		
Single	–	26 (65)
Married	–	12 (30)
Married, separated	–	2 (5)
Education		
Secondary school/ Vocational certificate	-	4 (10.00)
Bachelor’s degree	–	21 (52.5)
Higher than a bachelor’s degree	–	15 (37.5)
Household salary		
< 20,000 THB/month	–	6 (15)
20,000–49,999 THB/month	–	18 (45)
50,000–100,000 THB/month	–	13 (32.5)
> 100,000 THB/month	–	3 (7.5)
MEQ Group		
Morning type	–	1 (2.5)
Intermediate type	–	30 (75)
Evening type	–	9 (22.5)
Caffeine		
Not use	–	15 (37.5)
Use	–	25 (62.5)
Alcohol		
Not use	–	37 (92.5)
Use	–	3 (7.5)
Smoking		
Not use	–	39 (97.5)
Use	–	1 (2.5)
Sedative drug		
Not use	–	35 (87.5)
Use	–	5 (12.5)
DBAS	99.6 ± 22.13	–

MEQ, Morningness-Eveningness Questionnaire; DBAS, Dysfunctional Beliefs and Attitudes about Sleep

From sleep questionnaire data, the average ISI was 14.57, which was between the cut-off point of subthreshold and moderate severity. The average PSQI score was 11.32, which represented the poor sleep quality of the participants. From the sleep diary data, the baseline average sleep efficiency of participants was approximately 72% and total sleep time was approximately 6 hours. Sleep onset latency was the most prevalent problem (presented in 98% of the participants) and the most prolonged parameter when compared to the normal value (average sleep onset latency was 68 minutes). Early morning awakening was less prevalent (presented in 55% of the participants) and borderline prolonged when compared to the normal value (average early morning awakening duration was 34 minutes). Wake after sleep onset was present in only 15% of participants and the average duration of wake after sleep onset was still in the normal value (average wake after sleep onset duration was 18 minutes) (Tables 2, 3).

**Table 2:** Pre and post-sleep parameters.

Parameter	Pre [mean ± S.D. or median (IQR)]	Post [mean ± S.D. or median (IQR)]	Different [mean (95% CI)]	*p* value
Sleep efficiency (%)	71.94 ± 11.20	86.53 ± 7.02	14.58 (11.35, 17.80)	<0.001^*^
Total sleep time (hours)	6.07 ± 0.97	6.39 ± 0.91	0.31 (0.05, 0.57)	0.017^*^
Sleep onset latency (minutes)	68.21 (44.46, 90)	28.93 (18.43, 41.71)	−45.59 (−60.14, −31.03)	<0.001^*^
Wake after sleep onset (minutes)	17.60 (8.35, 26.6)	8.28 (4.29, 17.35)	−9.73 (−17.72, −1.74)	0.018*
Early morning awakening (minutes)	33.75 (19.82, 58.93)	9.64 (5, 19.64)	−31.81 (−20.32, −43.31)	<0.001^*^
Time in bed (hours)	8.5 ± 0.99	7.4 ± 0.82	−1.09 (−1.47, −0.72)	<0.001^*^
ISI	14.57 ± 3.49	8.7 ± 4.14	−5.87 (−7.37, −4.37)	<0.001^*^
ESS	6.5 (4, 9)	4 (2.5, 6)	−2.02 (−3.29, −0.75)	0.002^*^
PSQI	11.32 ± 2.86	6.55 ± 2.76	−4.77 (−5.81, −3.73)	<0.001^*^
Subjective sleep quality	2.92 ± 0.45	3.54 ± 0.72	0.57 (0.35, 0.79)	<0.001^*^

ISI, Insomnia Severity Index; ESS, Epworth Sleepiness Scale; PSQI, Pittsburgh Sleep Quality Index. ^*^ = *p* < 0.05.

**Table 3:** Pre and post-proportion of the participants with normal values for each sleep parameter.

Parameter	Pre [*n* (%)]	Post [*n* (%)]	*p* value
Sleep efficiency (%)			
< 85%	40 (100)	15 (37.5)	<0.001^*^
>/= 85%	0 (0)	25 (62.5)	
Total sleep time (hours)			
< 7	33 (82.5)	27 (67.5)	<0.001^*^
>/= 7	7 (17.5)	13 (32.5)	
Sleep onset latency (minutes)			
> 30	38 (98)	18 (45)	<0.001^*^
</= 30	2 (5)	22 (55)	
Wake after sleep onset (minutes)			
> 30	6 (15)	4 (10)	<0.001^*^
</= 30	34 (85)	36 (90)	
Early morning awakening (minutes)			
> 30	22 (55)	1 (2.5)	<0.001^*^
</= 30	18 (45)	39 (97.5)	
ISI			
>/= 8	40 (100)	26 (65)	<0.001^*^
< 8	0 (0)	14 (35)	
ESS			
> 10	5 (12.5)	3 (7.5)	<0.001^*^
</= 10	35 (87.5)	37 (92.5)	
PSQI			
>/= 6	40 (100)	24 (60)	<0.001^*^
< 6	0 (0)	16 (40)	

ISI, Insomnia Severity Index; ESS, Epworth Sleepiness Scale; PSQI, Pittsburgh Sleep Quality Index. ^*^ *= p* < 0.05.

During the 4-week intervention period, 40% of the participants went to bed within 30 minutes of the designated bedtime recommended by the Nitra application for ≥70% of all nights and 45% of the participants got up within 30 minutes of the designated wake-up time recommended by the Nitra application for ≥70% of all nights. In total, 58% of the participants read ≥50% of all the articles provided by the application.

There was a significant improvement in many parameters from the sleep diary when comparing before and after using the Nitra application. These included sleep efficiency [71.94 ± 11.20 vs. 86.53 ± 7.02 (mean difference 14.58; 95% CI 11.35–17.80, *p* < 0.001)], total sleep time [6.07 ± 0.97 vs. 6.39 ± 0.91 (mean difference 0.31; 95% CI 0.05–0.57, *p* = 0.017)], sleep onset latency [68.21 (44.46, 90) vs. 28.93 (18.43, 41.71) (mean difference − 45.59; 95% CI -60.14- -31.03, *p* < 0.001)], wake after sleep onset [17.60 (8.35, 26.6) vs. 8.28 (4.29, 17.35) (mean difference − 9.73; 95% CI -17.72- -1.74, *p* = 0.018)], and early morning awakening [33.75 (19.82, 58.93) vs. 9.64 (5, 19.64) (mean difference − 31.81; 95% CI -20.32- -43.31, *p* < 0.001)]. Time in bed was also significantly decreased [8.5 ± 0.99 vs. 7.4 ± 0.82 (mean difference − 1.09; 95% CI -1.47- -0.72, *p* < 0.001)] (Table 2).

For the other sleep parameters from the sleep questionnaire, there was also a significant improvement in ISI [(14.57 ± 3.49 vs. 8.7 ± 4.14 (mean difference − 5.87; 95% CI -7.37- -4.37, *p* < 0.001)], PSQI [(11.32 ± 2.86 vs. 6.55 ± 2.76) (mean difference − 4.77; 95% CI -5.81- -3.73, *p* < 0.001)], and average subjective sleep quality [(2.92 ± 0.45 vs. 3.54 ± 0.72) (mean difference 0.57; 95% CI -0.35-0.79, *p* < 0.001)] after using the Nitra application (Table 2).

When comparing the proportion of participants who had a normal value of each sleep parameter before and after using the Nitra application, there was a significant increase in the proportion of the participants who had normal sleep efficiency (≥ 85%) (0% vs. 62.5%, *p* < 0.001), total sleep time (≥ 7 hours) (17.5% vs. 32.5%, *p* < 0.001), sleep onset latency (≤ 30 minutes) (5% vs. 55%, p < 0.001), wake after sleep onset (≤ 30 minutes) (85% vs. 90%, *p* < 0.001), and early morning awakening (≤ 30 minutes) (45% vs. 97.5%, *p* < 0.001). The proportion of the participants who had normal values of ISI (<8) and PSQI (<6) also increased significantly (0% vs. 35%, *p* < 0.001 and 0% vs. 40%, *p* < 0.001, respectively) (Table 3).

When the evaluation was made to identify the factors associated with the change in sleep efficiency after using the Nitra application, no factors were shown to be significantly associated with the change in sleep efficiency (Table 4).

**Table 4:** Evaluation of the factors associated with sleep efficiency change after using the Nitra application.

Factor	Delta Post - Pre S.E	*p* value
Age		0.466
Sex		
Male [median (IQR)]	12 (10, 25.07)	0.570
Female [median (IQR)]	13.07 (7.07, 20.93)	
Marital status		
Single [median (IQR)]	12.57 (7.07, 20.93)	0.624
Married [median (IQR)]	13.68 (9.36, 22.54)	
Married, separated [median (IQR)]	9.89 (8.21, 11.57)	
Education		
Secondary school/ Vocational certificate [median (IQR)]	20.965 (6.57, 36.86)	0.362
Bachelor’s degree [median (IQR)]	13.22 (10, 21.79)	
Higher than bachelor’s degree (median (IQR)]	11.79 (4.85, 14.78)	
Household salary		
< 20,000 THB/month [median (IQR)]	15.745 (11.79, 26.28)	0.717
20,000–49,999 THB/month [median (IQR)]	13.18 (4.93, 21.79)	
50,000–100,000 THB/month [median (IQR)]	12 (9.07, 15.36)	
> 100,000 THB/month [median (IQR)]	13.57 (8.28, 25.07)	
MEQ Group		
Morning type [median (IQR)]	13.22 (13.22, 13.22)	0.567
Intermediate type [median (IQR)]	11.925 (6.07, 21.79)	
Evening type [median (IQR)]	13.14 (9.36, 20.93)	
Baseline ISI		0.360
Baseline ESS		0.124
DBAS		0.763
Baseline PSQI		0.087
Caffeine		
Not use [median (IQR)]	10 (6.07, 20.93)	0.342
Use (median (IQR)]	13.57 (9.36, 21.79)	
Alcohol		
Not use [median (IQR)]	12.13 (8.21, 21.79)	0.857
Use [median (IQR)]	13.07 (9.07, 14.29)	
Smoking		
Not use [median (IQR)]	12.13 (8.21, 20.93)	0.152
Use [median (IQR)]	31.43 (31.43, 31.43)	
Sedative drug		
Not use [median (IQR)]	12.13 (8.21, 21.79)	0.838
Use [median (IQR)]	13.57 (9.36, 18.86)	
Article read		
Read <50% of all articles [median (IQR)]	10 (4.93, 14.29)	0.250
Read >/= 50% of all articles [median (IQR)]	13.14 (9.36, 23.29)	
Bedtime within 30 minutes of the designated time		
< 70% of all bedtime [median (IQR)]	11.89 (5.5, 20.28)	0.171
>/= 70% of all bedtime [median (IQR)]	13.96 (10.46, 23.53)	
Wake-up time within 30 minutes of the designated time		
< 70% of all bedtime [median (IQR)]	12.67 (8.21, 23.29)	0.989
>/= 70% of all bedtime [median (IQR)]	12.46 (8.28, 18.86)	

MEQ, Morningness-Eveningness Questionnaire; ISI, Insomnia Severity Index; ESS, Epworth Sleepiness Scale; DBAS, Dysfunctional Beliefs and Attitudes about Sleep; PSQI, Pittsburgh Sleep Quality Index.

However, when the evaluation was made to identify the factors associated with achieving normal sleep efficiency after using the Nitra application, participants who had a low baseline ISI and went to bed and woke up within 30 minutes of the designated bedtime and wake-up time recommended by Nitra application for ≥70% of all nights demonstrated an increased chance of achieving normal sleep efficiency after using the Nitra application (Table 5). The odds ratio of each factor was 0.78 (95% CI 0.63–0.97), 4.33 (95% CI 0.97–19.20), and 1.34 (95% CI 1.34–26.80), respectively (Table 6).

**Table 5:** Evaluation of the factors associated with achieving normal sleep efficiency after using the Nitra application.

Factor	Post S.E. < 85%	Post S.E. >/= 85%	*p* value
Age (mean ± S.D.)	48.06 ± 12.98	44.36 ± 12.65	0.380
Sex			
Male [*n* (%)]	4 (26.67)	5 (20.00)	0.705
Female [*n* (%)]	11 (73.33)	20 (80.00)	
Marital status			
Single [*n* (%)]	9 (60.00)	17 (68.00)	0.266
Married [*n* (%)]	4 (26.67)	8 (32.00)	
Married, separated [*n* (%)]	2 (13.33)	0 (0)	
Education			
Secondary school/ Vocational certificate [*n* (%)]	3 (20.00)	1 (4.00)	0.362
Bachelor’s degree [*n* (%)]	7 (46.67)	14 (56.00)	
Higher than bachelor’s degree [*n* (%)]	5 (33.33)	10 (40.00)	
Household salary			
< 20,000 THB/month [*n* (%)]	2 (13.33)	4 (16.00)	1.000
20,000–49,999 THB/month [*n* (%)]	7 (46.67)	11 (44.00)	
50,000–100,000 THB/month [*n* (%)]	5 (33.33)	8 (32.00)	
> 100,000 THB/month [*n* (%)]	1 (6.67)	2 (8.00)	
MEQ Group			
Morning type [*n* (%)]	1 (6.67)	0	0.054
Intermediate type [*n* (%)]	13 (86.67)	17 (68.00)	
Evening type [*n* (%)]	1 (6.67)	8 (32.00)	
Baseline ISI (mean ± S.D.)	16.2 ± 3.02	13.6 ± 3.43	0.020^*^
Baseline ESS (mean ± S.D.)	6 ± 3.94	7.4 ± 3.14	0.223
DBAS (mean ± S.D.)	98.53 ± 18.05	100.24 ± 24.59	0.816
Baseline PSQI (mean ± S.D.)	11.2 ± 3.12	11.4 ± 2.76	0.834
Caffeine			
Not use [*n* (%)]	7 (46.67)	8 (32.00)	0.354
Use [*n* (%)]	8 (53.33)	17 (68.00)	
Alcohol			
Not use [*n* (%)]	15 (100.00)	22 (88.00)	0.279
Use [*n* (%)]	0 (0)	3 (88.00)	
Smoking			
Not use [*n* (%)]	15 (100.00)	24 (96.00)	1.000
Use [*n* (%)]	0 (0)	1 (4.00)	
Sedative drug			
Not use [*n* (%)]	13 (86.67)	22 (88.00)	1.000
Use [*n* (%)]	2 (13.33)	3 (12.00)	
Article read			
Read <50% of all articles [*n* (%)]	7 (46.67)	10 (53.33)	0.680
Read >/= 50% of all articles [*n* (%)]	8 (53.33)	15 (60.00)	
Bedtime within 30 minutes of designated time			
< 70% of all bedtime [*n* (%)]	12 (80.00)	12 (48.00)	0.046^*^
>/= 70% of all bedtime [*n* (%)]	3 (20.00)	13 (52.00)	
Wake up time within 30 minutes of designated time			
< 70% of all bedtime [*n* (%)]	12 (80.00)	10 (40.00)	0.014^*^
>/= 70% of all bedtime [*n* (%)]	3 (20.00)	15 (60.00)	

MEQ, Morningness-Eveningness Questionnaire; ISI, Insomnia Severity Index;

ESS, Epworth Sleepiness Scale; DBAS, Dysfunctional Beliefs and Attitudes about Sleep;

PSQI, Pittsburgh Sleep Quality Index. ^*^ = *p* < 0.05.

**Table 6:** The odds ratio of each factor associated with achieving normal sleep efficiency after using the Nitra application.

Factor	Odds ratio	95% CI	*p* value
Baseline ISI	0.78	0.63–0.97	0.028^*^
Bedtime within 30 minutes of the designated time			
< 70% of all bedtime [*n* (%)]	1		
>/= 70% of all bedtime [*n* (%)]	4.33	0.97–19.20	0.054
Wake-up time within 30 minutes of the designated time			
< 70% of all bedtime [*n* (%)]	1		0.019^*^
>/= 70% of all bedtime [*n* (%)]	6	1.34–26.80	

ISI, Insomnia Severity Index. ^*^ = *p* < 0.05.

## 4. Discussion

This is the first study that has evaluated the effectiveness of the first internet-based CBT-i in Thailand (Nitra application). The results from our study demonstrated that the Nitra application effectively improved participants’ insomnia in many aspects. For the primary outcome, sleep efficiency was significantly increased by approximately 15% after completing 4 weeks of internet-based CBT-i sessions using the Nitra application. This result approximated the result from a previous study conducted by Collin A Espie in 2012 (10), which showed that 6 weekly sessions of online CBT-i were able to significantly increase sleep efficiency by approximately 20% compared to the baseline before attending online CBT-i. The difference in sleep efficiency improvement between our study and the aforementioned study may be related to the duration of CBT-i therapy, in which a longer duration may result in a greater improvement in sleep efficiency. However, approximately two-thirds of the participants achieved normal sleep efficiency (sleep efficiency ≥85%) after using the Nitra application.

Average total sleep time after study completion increased by approximately 19 minutes from baseline. This borderline increase in total sleep time was likely due to the short CBT-i intervention period of our study (4 weeks). The protocol of sleep restriction in our study limited the extension of sleep time to 15 minutes per week if the sleep efficiency from the previous week was >85%. This protocol resulted in a maximum extension of sleep duration of 45 minutes after study completion. When compared to the previous study of Collin A Espie (10), which showed an increase of total sleep time of 39 minutes after 6 weekly sessions of online CBT-i, extending the duration of the intervention period would likely result in a further increase in total sleep time.

Sleep onset latency, wake after sleep onset, and early morning awakening duration were significantly improved after completing the study. However, nearly half of the participants (45%) still had a sleep onset latency of longer than 30 minutes even though this was significantly decreased from the baseline (98%). For wake after sleep onset and early morning awakening, only a small number of participants (10 and 2%, respectively) still experienced these sleep parameters longer than 30 minutes. However, in our study, sleep onset latency was calculated by the duration between the time when the participants went to bed and the estimated time when the participants fell asleep, which may be overestimated due to some proportion of the time may not be intended to sleep (e.g., using a cellphone or watching TV during being on the bed). This limitation may have overestimated the baseline severity and underestimated the improvement of sleep onset insomnia in our study.

ISI, PSQI, and average subjective sleep quality showed significant improvements. However, only a minority of the participants (25%) achieved a normal ISI (< 8) after using the Nitra application. For PSQI, nearly half of the participants (40%) achieved a normal PSQI (< 6) after completing the intervention period.

When evaluated for the factors associated with achieving normal sleep efficiency after using the Nitra application, lower baseline ISI was associated with a higher chance of achieving normal sleep efficiency after completing the intervention. This finding was not surprising because a lower baseline ISI represented a lower insomnia severity, which would have a higher chance of being resolved after attending CBT-i. This finding also supported that the Nitra application could also be applicable to a non-insomnia population who has the tendency to develop insomnia. Compliance with the designated bedtime and wake-up time recommended by the Nitra application (which was defined by a participant’s actual bedtime and wake-up time being within 30 minutes of the designated bedtime and wake-up time by Nitra application for ≥70% of all nights) was also a predictor for a higher chance of achieving normal sleep efficiency (≥ 85%) after using the Nitra application. These findings supported the current evidence that the sleep restriction technique is one of the most effective techniques of CBT-i (25).

Nevertheless, there were some limitations in our study. First, the short intervention period (4 weeks) may have limited improvement of both the objective and subjective parameters of insomnia in our study. We hypothesize that extending the period of using the Nitra application may result in a further increase in sleep efficiency, total sleep time, and the proportion of participants resolving their insomnia. Second, sleep onset latency and early morning awakening in our study may be overestimated. A more detailed version of a sleep diary that can record the starting time in bed, the time intended to sleep, the time intended to wake up, and the wake-up time may reflect more accurate sleep onset latency and early morning awakening. Third, the number of articles read by each participant was recorded only when the participants manually clicked the button at the end of the article to confirm they had finished reading. This may have caused an underestimation of the number of articles read by each participant. Automatic article reading tracking may result in more accurate information. Finally, the exclusion of participants who had normal sleep efficiency (≥ 85%) may limit the generalizability of our findings to people with insomnia by ICSD-3 criteria but failed to include self-reported sleep deficits. Less stringent inclusion and exclusion criteria for participants in the future study may make more generalizability of the intervention for patients with insomnia symptoms. In conclusion, the Nitra application effectively improved participants’ sleep efficiency and other parameters in chronic subthreshold to moderate insomnia. Future study with a longer intervention period, automatic data tracking, and more detailed sleep diary record is needed to further study the efficacy of the Nitra application.

## Data availability statement

The raw data supporting the conclusions of this article will be made available by the authors, without undue reservation.

## Ethics statement

The studies involving human participants were reviewed and approved by Institutional Review Board, Faculty of Medicine, Chulalongkorn University. The patients/participants provided their written informed consent to participate in this study.

## Author contributions

WT, KT, DM, NC, and PS conceptualized, validated, and wrote the original draft of the manuscript. WT conducted the study. WT, DM, NC, and PS analyzed the data of the study. All authors contributed to the article and approved the submitted version.

## Funding

This research was financially supported by the Ratchadaphiseksomphot Endowment Fund of Chulalongkorn University. The creation of the Nitra application was funded by the endowment fund of the Excellence Center for Sleep Disorders, King Chulalongkorn Memorial Hospital, Thai Red Cross Society (#GA65/13).

## Conflict of interest

The authors declare that the research was conducted in the absence of any commercial or financial relationships that could be construed as a potential conflict of interest.

## Publisher’s note

All claims expressed in this article are solely those of the authors and do not necessarily represent those of their affiliated organizations, or those of the publisher, the editors and the reviewers. Any product that may be evaluated in this article, or claim that may be made by its manufacturer, is not guaranteed or endorsed by the publisher.

## Abbreviations

ISI: Insomnia severity index
ESS: Epworth Sleepiness Scale
MEQ: Morningness-Eveningness questionnaire
PSQI: Pittsburgh Sleep Quality Index
DBAS: Dysfunctional Beliefs and Attitudes about Sleep
